# Transcriptional Profiling of SARS-CoV-2-Infected Calu-3 Cells Reveals Immune-Related Signaling Pathways

**DOI:** 10.3390/pathogens12111373

**Published:** 2023-11-20

**Authors:** Eric Petterson Viana Pereira, Stela Mirla da Silva Felipe, Raquel Martins de Freitas, José Ednésio da Cruz Freire, Antonio Edson Rocha Oliveira, Natália Canabrava, Paula Matias Soares, Mauricio Fraga van Tilburg, Maria Izabel Florindo Guedes, Chad Eric Grueter, Vânia Marilande Ceccatto

**Affiliations:** 1 Superior Institute of Biomedical Sciences, State University of Ceará, Fortaleza 60714-903, CE, Brazil; stelamirla@gmail.com (S.M.d.S.F.); raquel.martins.rf@gmail.com (R.M.d.F.); jednesio@gmail.com (J.E.d.C.F.); paula.soares@uece.br (P.M.S.); 2 Experimental Biology Center, University of Fortaleza, Fortaleza 60811-905, CE, Brazil; oliveiraaer@unifor.br; 3 Biotechnology and Molecular Biology Laboratory, State University of Ceará, Fortaleza 60714-903, CE, Brazil; canabravanatalia@gmail.com (N.C.); mauricio_van_tilburg@yahoo.com.br (M.F.v.T.); florinfg@uol.com.br (M.I.F.G.); 4 Department of Internal Medicine, University of Iowa, Iowa City, IA 52242, USA; chad-grueter@uiowa.edu

**Keywords:** COVID-19, Calu-3 cells, host-pathogen interaction, RNA-seq, transcriptome

## Abstract

The COVID-19 disease, caused by the Severe Acute Respiratory Syndrome Coronavirus 2 (SARS-CoV-2), emerged in late 2019 and rapidly spread worldwide, becoming a pandemic that infected millions of people and caused significant deaths. COVID-19 continues to be a major threat, and there is a need to deepen our understanding of the virus and its mechanisms of infection. To study the cellular responses to SARS-CoV-2 infection, we performed an RNA sequencing of infected vs. uninfected Calu-3 cells. Total RNA was extracted from infected (0.5 MOI) and control Calu-3 cells and converted to cDNA. Sequencing was performed, and the obtained reads were quality-analyzed and pre-processed. Differential expression was assessed with the EdgeR package, and functional enrichment was performed in EnrichR for Gene Ontology, KEGG pathways, and WikiPathways. A total of 1040 differentially expressed genes were found in infected vs. uninfected Calu-3 cells, of which 695 were up-regulated and 345 were down-regulated. Functional enrichment analyses revealed the predominant up-regulation of genes related to innate immune response, response to virus, inflammation, cell proliferation, and apoptosis. These transcriptional changes following SARS-CoV-2 infection may reflect a cellular response to the infection and help to elucidate COVID-19 pathogenesis, in addition to revealing potential biomarkers and drug targets.

## 1. Introduction

The COVID-19 pandemic has become a global health crisis with profound implications for public health and the economy. Since its initial outbreak in late 2019, the virus has rapidly spread across continents, infecting millions of individuals and causing significant deaths. The World Health Organization (WHO) declared COVID-19 a pandemic on 11 March 2020, reflecting the unprecedented scale and severity of this disease that, even after the discontinuation of the Public Health Emergency of International Concern, is still a major threat [1]. This health crisis has underscored the urgent need to deepen our understanding of the virus and its mechanisms of infection to develop effective prevention strategies, diagnostics, and therapeutics [1,2].

In this context, the Calu-3 cell line has emerged as a valuable in vitro model for studying the SARS-CoV-2 virus and its implications in COVID-19. Calu-3 cells are derived from human lung adenocarcinoma and exhibit characteristics that closely resemble airway epithelial cells, which make them a biologically relevant model for respiratory infections [3]. These cells express the angiotensin-converting enzyme 2 (ACE2) receptor and dipeptidyl peptidase-4 (DPP-4), which serve as primary entry points for SARS-CoV-2 into host cells [4], and the proteases TMPRSS2 and furin, required for the cleavage and activation of the Spike (S) protein [5]. Such features allow for efficient viral infection and replication, making Calu-3 cells a suitable model for the analysis of SARS-CoV-2 replication dynamics, host immune responses, and drug efficacy [6]. Furthermore, Calu-3 cells form polarized monolayers with functional cilia and mucus production, replicating critical features of the respiratory epithelium and enabling the investigation of viral transmission and pathogenesis in the context of the airway microenvironment [7].

Calu-3 cells have provided insights into the viral cycle, virus-induced host immune responses, and the identification of potential therapeutic targets [2,8]. Consequently, these cells have been employed to evaluate antiviral compounds, assess the neutralizing capacity of antibodies, and study the effect of viral mutations on infectivity [9,10], further evidenced by their compatibility with high-throughput screening assays, facilitating the identification of novel antiviral agents and the evaluation of drug repurposing strategies [11].

The study of transcriptomes provides a comprehensive snapshot of gene expression and reflects the dynamic activity of genes within a cell and the molecular mechanisms underlying SARS-CoV-2 viral infections. Analyzing the transcriptome, researchers can identify genes differentially expressed upon viral infection, unravel signaling pathways modulated during infection, and gain insights into the host’s immune response to the virus. By comprehensively characterizing the transcriptomic changes induced by viral infections, we can enhance our understanding of the intricate interplay between the virus and host cells, leading to valuable insights into the pathogenesis of COVID-19 [12]. So far, transcriptome analysis of SARS-CoV-2-infected cells has generally shown cellular responses related to IFN signaling, innate immunity, inflammation, and defense against virus [13,14,15,16,17]. Although these responses constitute hallmarks of viral infections, the activation degree of functional categories and signaling pathways can vary [18]. Moreover, differences in the infection protocols and in the time between infection and cell lyses can lead to distinct results.

This work sought to analyze changes in the transcriptome of Calu-3 cells in response to 24 h SARS-CoV-2 infection, understand the mechanisms of immune response to viral infection, and reveal potential drug targets or biomarkers that could be used in the diagnosis or prognosis of COVID-19.

## 2. Materials and Methods

### 2.1. Virus and Cells

The SARS-CoV-2 strain (Wuhan/B.1.212) was obtained from the nasopharyngeal swab of a COVID-19 patient diagnosed by RT-qPCR at the Hematology and Hemotherapy Center of Ceará (HEMOCE) in Fortaleza, Brazil. The virus was isolated from Vero E6 cells as described by Harcourt et al. [19] and sequenced by the Genomic Surveillance Network of the Oswaldo Cruz Foundation (Fiocruz-Ce) using a COVIDSeq^TM^ kit (Illumina, San Diego, CA, USA). Calu-3 cells were obtained from the Rio de Janeiro Cell Bank (BCRJ—Rio de Janeiro, Brazil) and cultured in Dulbecco Modified Eagle Medium (DMEM; VitroCell, Campinas, SP, Brazil) supplemented with 1% penicillin–streptomycin and 20% Fetal Bovine Serum (FSB; VitroCell, Campinas, SP, Brazil) at 37 °C in a 5% CO_2_ incubator, according to the supplier’s instructions. The cells were confirmed free of mycoplasma contamination and authenticated based on their morphology and growth characteristics. Calu-3 is not listed as a commonly misidentified cell line according to the International Cell Line Authentication Committee (ICLAC) [20].

### 2.2. Infection and RNA Extraction

Approximately 1 × 10^6^ Calu-3 cells/well were seeded into 6-well plates and infected with SARS-CoV-2 at a multiplicity of infection (MOI) of 0.5. Briefly, the infection was performed in unsupplemented DMEM, and the infected cells were maintained at 37 °C in a 5% CO_2_ incubator with gently stirring every 15 min. 1 h postadsorption, the inoculums were discarded, the wells were rinsed with DMEM (37 °C), and the cells were maintained in supplemented DMEM, as previously described. Culture medium was used as a negative control, and each group was performed in quintuplicate. 24 h postinfection, the cells were lysed using TRIZol (Invitrogen, Waltham, MA, USA), and total RNA extraction was performed using a PureLink^TM^ RNA mini kit (Invitrogen, Waltham, MA, USA). The RNA was further treated with DNAse I (TransGen Biotech, Beijing, China) at 37 °C for 30 min (1 U of DNase I per μg of RNA) and cleaned up using an RNeasy Mini kit (Qiagen, Hilden, Germany). Treated RNA was recovered in 30 μL of nuclease-free water and subsequently utilized for cDNA synthesis. The synthesis of cDNA samples was carried out according to the recommendations provided by the manufacturer of the High Capacity cDNA Reverse Transcription Kit (Applied Biosystems, Foster City, CA, USA). The reactions were conducted using a Aeris^TM^ thermocycler (ESCO^®^ Lifesciences, Singapore) with the following cycling parameters: an initial step of 10 min at 25 °C, followed by 120 min at 37 °C, 5 min at 85 °C, and 5 min at 4 °C. The concentrations of nucleic acids in both RNA and cDNA samples were determined using an appropriate microplate reader (Biotek Synergy™ HTX Multi-Mode Reader, Winooski, VT, USA), and the samples were stored at −20 °C.

### 2.3. Library Construction and Next-Generation Sequencing

Quality control of the samples was performed using an Agilent 2100 Bioanalyzer (Agilent Technologies, Palo Alto, CA, USA). The cDNA was randomly fragmented, and libraries were prepared using the Illumina TruSeq Nano DNA Library Prep Kit according to the manufacturer’s instructions. All libraries were sequenced in paired-end mode (151-bp reads) on the NovaSeq 6000 platform (Illumina, San Diego, CA, USA). The raw sequencing data were deposited in NCBI under BioProject submission (PRJNA993611).

### 2.4. RNA-Seq Data Analysis

#### 2.4.1. Processing and Filtering

The reads were pre-processed using the Fastp tool to trim adapters and remove low-quality sequences with a Phred quality score < 20 [21]. FastQC was used to evaluate the quality of the sequences before and after pre-processing [22]. The processed reads were aligned to the human genome GRCh38.p13, version 106 (61,552 genes), using the STAR aligner [23]. The aligned paired-end reads were quantified using Feature Counts, with the following parameters: –B (R1 and R2 reads), -C (fragments with the paired reads mapped to different chromosomes are not accounted), -g *‘gene id’*(mapping to genes), -s 0 (non-strand-specific counting), and - - *extra Attributes ‘gene_name’, ‘gene_biotype’*(gene annotation). These settings were used to obtain the raw data according to Liao et al. [24].

#### 2.4.2. Determination of Differentially Expressed Genes (DEGs)

The R-package EdgeR was used for several analyses, including filtering low-expression genes, evaluating sample reproducibility, and identifying DEGs, as recommended by Robinson et al. [25]. Low-expression genes featuring a count per million (CPM) value < 1 in at least 3 samples were excluded from the analyses. Outlier samples were identified and removed based on Principal Component Analysis (PCA), Multidimensional Scaling (MDS), and Euclidean clustering. DEGs were selected based on specific criteria: Log2Fold Change(FC) ≥ 0.5 or ≤−0.5; and *p*-value ≤ 0.05.

### 2.5. Functional Enrichment Analysis

The Gene Ontology (GO) enrichment analysis of the DEGs was conducted to investigate their association with biological processes, molecular functions, and cellular components. Additionally, pathway enrichment analysis was performed using EnrichR to identify enriched biological pathways among the DEGs [26] using the Kyoto Encyclopedia of Genes and Genomes (KEGG) and WikiPathways. The functional enrichment analysis was performed separately for the up- and down-regulated DEGs, and an adjusted *p*-value of ≤0.05 was set as statistically significant.

### 2.6. Functional Enrichment Analysis

All the statistical analyses were performed in R (4.2.2). The differential expression analysis was conducted using Student’s *t*-test. To account for multiple testing, the Benjamini–Hochberg procedure was employed for false discovery rate (FDR) correction.

## 3. Results

### 3.1. Transcriptional Profile of SARS-CoV-2-Infected Cells

To characterize the transcriptional response in SARS-CoV-2-infected cells, the total RNA was collected from infected and uninfected Calu-3 cells. The transcriptomes of these samples were determined by using high-throughput RNA sequencing, which provides a comprehensive view of gene expression at a genome-wide level. After pre-processing and quality filtering, the reads were mapped to the human reference genome and quantified. The identification of the DEGs was performed after filtering for genes with low expression and evaluating sample reproducibility. Robust and reproducible data were obtained after removing outlier samples. Two samples of each group were excluded as outliers. A total of 171,095,226 reads were obtained: 89,826,138 from the infected group and 81,269,088 from the control group. For each sample, >90% of the reads displayed a Phred quality score ≥ 30 (Q30). A Phred score of 30 indicates the possibility of 1 erroneous base for every 1000 bp sequenced. We found 14,399 genes that were expressed in Calu-3 cells, of which 1040 (7.22%) were differentially expressed (Supplementary File S1). Furthermore, differences were observed between the infected and control samples in the Principal Component Analysis (PCA). Two groups were found to be located at opposite ends of the coordinate axis, particularly in terms of the principal component 1 (PC1), and possibly also in the principal component 2 (PC2) (Supplementary File S2). Indeed, the observed separation of the infected and control groups in the PCA plot is highly indicative of the impact of SARS-CoV-2 infection on the transcriptional profiles of Calu-3 cells. This feature reflects the distinct gene expression patterns associated with the viral infection and highlights the transcriptional changes induced by SARS-CoV-2. The clear differentiation between the infected and control samples in the PCA plot provides strong evidence for the influence of SARS-CoV-2 infection on the cellular transcriptome.

### 3.2. Differentially Expressed Genes (DEGs)

**Figure 1:**
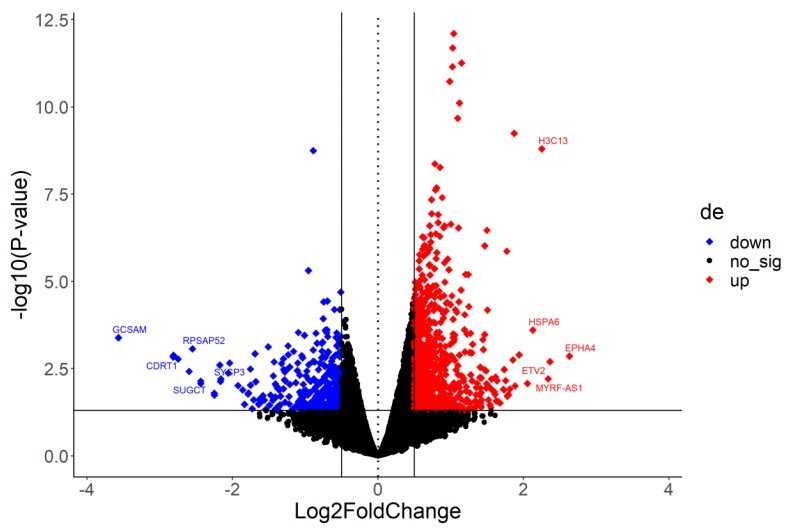
Volcano plot showing differentially expressed genes (DEGs) in SARS-CoV-2-infected Calu-3 cells vs. control (uninfected) cells. The most expressive DEGs are identified. de: Differential expression. down: Down-regulated. no_sig: Non-significant. up: Up-regulated.

**Figure 2:**
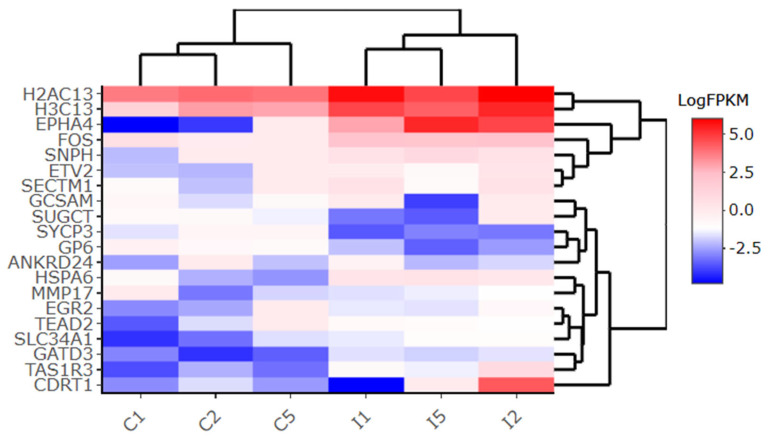
Heatmap of the top 20 DEGs in Calu-3 cells. Expression patterns of genes are compared between control (C1, C2, and C5) and infected (I1, I2, and I5) samples. For each gene, the relative values of gene expression are depicted in a blue-red scale, in which red tones are representative of higher expression, and blue tones, of lower expression. FPKM: Fragments per Kilobase Million.

DEGs were identified from the control (uninfected) vs. SARS-CoV-2-infected groups (*n* = 3), following the parameters Log_2_fc ≥ 0.5 or ≤−0.5 and *p* ≤ 0.05. These DEGs were then employed for functional annotation. Overall, 1040 DEGs were significantly regulated in 24 h post-infection Calu-3 cells, with 695 being up-regulated and 345 being down-regulated (Supplementary File S1). In Figure 1, all DEGs are displayed in a volcano plot. A heatmap of the top 20 DEGs was obtained (Figure 2), and the genes with the most expressive differential expression values are detailed in a top 20 table (Table 1). The highly up-regulated DEGs include the EPH receptor A4 (EPHA4; log_2_fc 2.631), ETS variant transcription factor 2 (ETV2; 2.364), and H3 clustered histone 13 (H3C13; 2.252) genes. In turn, the main down-regulated DEGs include the germinal center associated signaling and motility (GSAM; log_2_fc −3.567), F-box and WD repeat domain containing 10B (CDRT1; −2.814), and Succinyl-CoA:glutarate-CoA transferase (SUGCT; −2.436) genes. In addition, non-coding RNA genes were also differentially expressed in infected vs. uninfected Calu-3 cells. Out of the 695 up-regulated DEGs, 71 are related to non-coding RNAs, while 70 non-coding RNAs, out of 345 DEGs, were found among down-regulated genes. In both cases, lncRNA was the major class of non-coding RNAs found in our samples.

### 3.3. Functional Enrichment Analysis

#### 3.3.1. Gene Ontology

**Figure 3:**
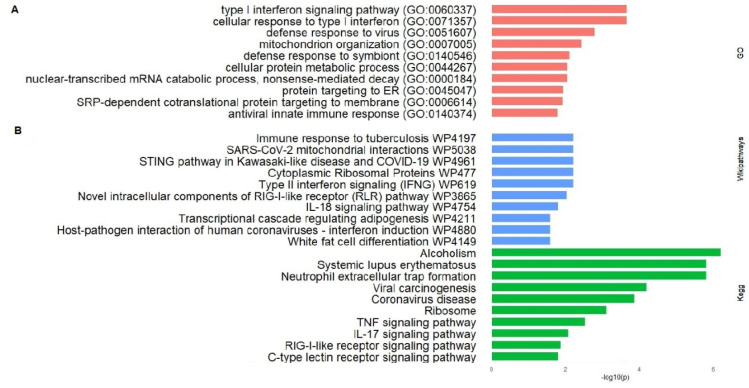
Functional enrichment of up-regulated DEGs. Enriched terms for Gene Ontology’s (GO) biological process (**A**). Enriched terms for KEGG Pathways and WikiPathways (**B**).

The GO enrichment of DEGs was divided into three categories: biological process (BP), cellular component (CC), and molecular function (MF). The top 10 terms for BP are shown in Figure 3A. For the up-regulated DEGs, the most enriched BP terms were cellular response to type I interferon, type I interferon signaling pathway, and defense response to virus. Additionally, there were also significantly enriched BP terms (*p*-adjust. ≤ 0.05) related to mitochondrion organization, cellular protein metabolic process, protein targeting to ER, chromatin assembly, and apoptotic process. The most enriched CC term was ribosome (*p*-adjust. 0.04). However, no significant MF term was achieved, with nucleosomal DNA binding (*p*-adjust. 0.33) being the most enriched. In turn, for the down-regulated genes, no significantly enriched term was identified for any of the three categories (BF, CC, and MF).

#### 3.3.2. Pathways Enrichment

The pathways’ enrichment of DEGs was performed, and the top 10 enriched pathways for KEGG and WikiPathways are displayed in Figure 3B. The most significant up-regulated KEGG pathways included terms like neutrophil extracellular trap formation (*p*-adjust. ≤ 0.01), systemic lupus erythematosus (*p*-adjust. ≤ 0.01), coronavirus disease (*p*-adjust. ≤ 0.01), ribosome (*p*-adjust. ≤ 0.01), TNF signaling pathway (*p*-adjust. ≤ 0.01), IL-17 signaling pathway (*p*-adjust. ≤ 0.01), and RIG-I-like receptor signaling pathway (*p*-adjust. 0.01) (Figure 3B). In a similar analysis using enriched WikiPathways terms, we identified the following significant pathways: Type II interferon signaling (*p*-adjust. ≤ 0.01), cytoplasmic ribosomal proteins (*p*-adjust. ≤ 0.01), STING pathway in Kawasaki-like disease and COVID-19 (*p*-adjust. ≤ 0.01), SARS-CoV-2 mitochondrial interaction (*p*-adjust. ≤ 0.01), novel intracellular components of RIG-I-like receptor (RLR) pathway (*p*-adjust. ≤ 0.01), IL-18 signaling pathway (*p*-adjust. 0.01), and host–pathogen interaction of human coronaviruses-interferon induction (*p*-adjust. 0.02) (Figure 3B). In the pathway analysis of down-regulated DEGs, no significantly enriched KEGG term was identified. However, in the analysis of WikiPathway, one significantly enriched term was found, which is ciliopathies (*p*-adjust. ≤ 0.01).

## 4. Discussion

In this study, we performed the RNA-sequencing of Calu-3 cells 24 h after infection with SARS-CoV-2 (0.5 MOI). A total of 1040 DEGs—695 up- and 345 down-regulated—were found by applying a log2fc ≥ 0.5 and a *p*-value ≤ 0.05, with a non-stringent FDR. This approach enabled us to achieve enriched pathways related to SARS-CoV-2 infection that were reproducible between several databases and coherent with Gene Ontology, giving a broader view of the cellular response to infection. However, the more significant enriched pathways were also achieved when a stringent FDR ≤ 0.05 cut-off was applied. This straightforward approach of fold-change ranking combined with a non-stringent *p*-value threshold yielded reproducible lists of genes for both RNA-seq and microarray gene expression analyses [27,28].

The top 20 DEGs included up-regulated genes like EPHA4, HSPA6, SECTM1, FOS, and MMP17 (Table 1). The EPHA4 gene encodes a member of the ephrin receptor family [29]. This family is expressed in several tissues and organs, such as the lung, kidney, and heart, and is differentially expressed in human cancers [30]. It was found to promote cancer progression, angiogenesis, and neurodegeneration [31]. EPH receptors have been described as a possible receptor for SARS-CoV-2 entry in the central nervous system [32], and the receptor binding motif (RBM) of SARS-CoV-2 was recently described to mimic ephrin-a5 and -b2, which bind to EPHA4, with similar affinity values for the RBM-EPHA4 and ephrin-a5-EPHA4 bindings in a protein docking study [33]. This poses a challenge regarding whether the SARS-CoV-2 RBM can bind to EPH receptors in several tissues and trigger downstream signaling pathways related to cancer or to COVID-19 complications like inflammation, atherosclerotic plaque formation, or neurological sequelae [31,32].

In turn, the HSPA6 gene encodes an isoform of the heat shock protein 70 (HSP70) [34]. Heat shock proteins are stress-responsive proteins that deal with proteotoxic stresses, like heat, cold, microbial infections, and UV radiation [34,35,36,37]. They are also referred to as molecular chaperones and are involved in the folding, transport, assembly, and degradation of proteins [38,39]. The virus-induced overexpression of the host’s chaperones presents a dual role. It can both display an antiviral activity by stimulating an antiviral immune response or promoting infected cells’ death, and favor the virus life cycle by promoting the nuclear translocation of the viral genome and its replication and transcription events, as well as the synthesis of structural proteins and viral assembly and release [40]. Moreover, the up-regulation of chaperones can also counteract the ER stress through the unfolded protein response (UPR), allowing for a cytoprotective effect while enhancing the folding of viral proteins or the degradation of misfolded accumulated proteins [40]. The HSPA6 gene is considered a hub gene in SARS-CoV-2 infection and can differentiate remdesevir-treated COVID-19 patients [41]. It was also the most over-expressed host’s gene in transfected 293T cells expressing the SARS-CoV-2 ORF3A protein [42].

The SECTM1 gene encodes a Golgi-associated secreted and transmembrane protein mainly expressed in leucocytes and breast cancer cell lines. It displays broad structural features that resemble cytokines and growth factors [43] and is considered a potential activator of NF-κB [44]. The expression of SECTM1 was increased in airway epithelial cells from mice lungs infected with pneumococcal pneumonia. This overexpression took place at the early stages of infection and was type-I IFN- and STAT1-dependent. In this study, the SECTM1 protein was found to target neutrophils at the infected lungs and increase their expression of the neutrophil-attracting cytokine CXCL2, hence functioning as an epithelial product that favors a positive feedback loop of neutrophilic inflammation into lung tissue [45]. In addition, the SECTM1 protein was detected in respiratory epithelium cultures infected with the human Respiratory Syncytial Virus (hRSV), but not in the uninfected cultures [46]. The FOS gene encodes a transcription factor that is part of the AP-1 complex, implicated in carcinogenesis [47] and inflammatory antiviral signaling [46]. Its targeting by repurposing drugs was found to reduce the SARS-CoV-2 cytopathic effect (CPE) [48]. The MMP17 gene encodes a membrane-type member of the matrix metalloproteinase family (MMPs) with pro-TNF-α convertase activity [49].

**Figure 4:**
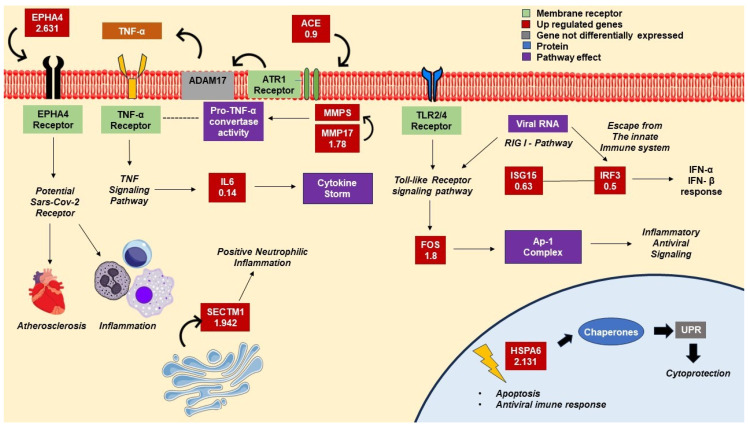
Calu-3 cells 24 h after SARS-CoV-2 infection. The transcriptional features of the infected cells indicate a metabolic model with the activation of inflammatory and antiviral signaling, in addition to both apoptotic and cytoprotective/proliferative signaling. Up-regulated genes include a potential SARS-CoV-2 receptor (EPHA4), a chaperone (HSPA6), pro-inflammatory transcription factors (IRF3 and FOS), and inflammatory mediators (ACE, MMP17, IL6, SECTM1, and ISG15).

Moreover, the functional enrichment of DEGs showed the predominance of genes involved in innate immune response, defense response to virus, and translation. The GO analysis revealed, for the BP category, enriched terms like cellular response to type I interferon, type I interferon signaling pathway, defense response to viruses, protein metabolic process, and chromatin assembly (Figure 3A). KEGG analysis showed the enrichment of pathways involved in neutrophil extracellular trap (NETs) formation, systemic lupus erythematosus (SLE), viral carcinogenesis, and COVID-19 disease. Immune-related pathways, like TNF, IL-17, RIG-I-like receptor, and C-type lectin receptor signaling pathways were also significantly enriched in KEGG analysis (Figure 3B). Coherently, WikiPathways analysis showed enriched terms like type II interferon signaling, cytoplasmic ribosomal proteins, STING pathway in Kawasaki-like disease and COVID-19, immune response to tuberculosis, novel intracellular components of RIG-I-like receptor pathway, IL-18, and host–pathogen interaction of human coronaviruses-interferon induction. Thus, in a framework of 24 h postinfection, our results are suggestive of a metabolic model mainly characterized by the activation of inflammatory and antiviral signaling, as summarized in Figure 4. Moreover, both apoptotic and cytoprotective/proliferative signaling may also be activated. As discussed below, such transcriptional signature may be prompted by several mechanisms and can be involved in COVID-19 pathogenesis.

### 4.1. Neutrophil Extracellular Trap Formation (NETs) and Autoimmunity

NETs formation (hsa04613) and SLE (hsa05322) KEGG pathways were significantly enriched in infected vs. uninfected Calu-3 cells. Both pathways contain mainly canonical core histone genes, like several H2A, H2B, H3, and H4 members. Histone octamers bind to DNA and form the nucleosome, the basic structure of chromatin. Core histones are replication-dependent clustered genes, and their expression only occurs during the S phase of the cell cycle [50,51]. Histones are the major component of neutrophil extracellular traps (NETs), which consist of network-like antimicrobial structures bearing nuclear proteins, DNA, and cytotoxic enzymes that capture and kill microorganisms [52,53]. This mechanism is part of the innate immune response and can be triggered by pro-inflammatory cytokines, LPS, intracellular ROS, microorganisms, and chemical agents in a process called NETosis [52]. The extracellular exposure of nuclear antigens following NETosis can elicit autoantibodies towards DNA and nuclear proteins, which are present in autoimmune diseases like SLE [54,55]. The concentration of NETs is increased in plasma, tracheal aspirate, and lung autopsies of COVID-19 patients [56], and is likely to play a key role in lung inflammatory damage and pulmonary microthrombiformation [57,58]. Nonetheless, this process is typical to immune cells and is unlikely to have taken place in our samples. Instead, the up-regulation of the core histones mRNA expression in SARS-CoV-2-infected Calu-3 cells is perhaps related to the cell cycle and proliferation, with cells entering the S phase [50,51].

However, the gene GSDMD was also significantly up-regulated in the KEGG pathway hsa04613. This gene encodes gasdermin D (GSDMD), a pore-forming protein that, upon activation by caspases and inflammasomes, triggers pyroptosis, a form of programmed cell death that features membrane pores and massive leakage of cytosolic content, including IL-1β and IL-18 [59]. By inducing a strong inflammatory response that constitutes a host’s immune defense mechanism, pyroptosis can lead to inflammatory diseases and autoimmunity [59]. Emerging evidence suggests that pyroptosis is linked to the inflammatory process in respiratory diseases and may lead to tissue injury and airway damage [60]. Sun et al. [61] showed that SARS-CoV-2 NSP6 induces pyroptosis in lung epithelial cells by impairing lysosome acidification, upstream of caspase-1 activation. In this study, NSP6-induced pyroptosis was prevented by both the knockdown of GSDMD and the pharmacological inhibition of caspase-1, which reduced GSDMD cleavage and activation. Furthermore, in an LPS-induced Acute Lung Injury (ALI) mice model, the inhibition of GSDMD by disulfiram prevented pyroptosis and alleviated ALI [62]. Interestingly, this gene was also significantly enriched in the WikiPathways STING pathway in Kawasaki-like disease and COVID-19 (WP4961). Indeed, the over-expression of several pro-apoptotic proteins, including GSDMD, have been described in patients with Kawasaki-like disease [63], an autoimmune condition that has been associated with COVID-19 [64].

### 4.2. Viral Carcinogenesis and Immune-Evasion

The KEGG pathway viral carcinogenesis (hsa05203) is significantly enriched in infected Calu-3 cells, which was also reported in a previous study [65]. Viruses are obligatory intracellular parasites that reprogram host cells’ signaling pathways that control cell death, proliferation, differentiation, genomic integrity, and recognition by the immune system; whose dysregulation may lead to the proliferation of aberrant cells [66]. Indeed, it is estimated that 10–15% of human cancers have a viral etiology [67,68], and SARS-CoV-2 is also able to modulate oncogenic signaling pathways [69]. The viral carcinogenesis pathway contains host proteins known to interact with proteins from well-established oncoviruses, such as hepatitis B/C virus, HPV, and HTLVI. Among the over-expressed genes belonging to this pathway, the EGR2 gene encodes a transcription factor that up-regulates the expression of Fas Ligand (FasL) [70]. The Fas receptor is constitutively expressed in human airway epithelium and triggers apoptosis when engaged by FasL [71], while the latter is constitutively expressed in few tissues and constitutes a mechanism of immune privilege by triggering apoptosis in Fas-expressing inflammatory cells [72]. FasL is overexpressed in lung carcinoma cells, in which it contributes to both immune evasion—by inducing the apoptosis of tumor-reactive T cells—and invasion—by killing surrounding Fas-positive cells [73]. Moreover, FasL can be over-expressed in circumstances of chemical and infectious insults and is likely to play a critical role in pulmonary inflammation and injury. The apoptosis of epithelial cells and alveolar macrophages mediated by Fas-Fas-L signaling, in addition to tissue injury, results in the release of IL-1β and chemokines, leading to neutrophil infiltration [71]. Such a mechanism was found to be involved in the pathogenesis of acute lung injury (ALI) and acute respiratory distress syndrome (ARDS) [74].

Other genes significantly up-regulated in the hsa05203 pathway were IRF3 and IRF7, which encode interferon regulatory factors that play key roles in the innate immune response to virus infection and the transcriptional activation of type-I IFN genes [75]. IRF3 displays antiviral activity [75] and is inhibited by proteins of several viruses with oncogenic potential, like cytomegalovirus [76] and HCV [77]. Conversely, the IRF7 gene was found to repress transcriptional activation by IFN and IRF1 and to support the latency of the Epstein–Barr virus (EBV) [78]. The phosphorylation and activation of the IRF3 protein are constrained by some SARS-CoV-2 proteins—NSP3, NSP6, NSP13, and ORF6—through the inhibition of the upstream RIG-I- and MDA5-IRF3/7 signaling [79]. Hence, the over-expression of the IRF3 mRNA in our samples could be a cellular response to counteract such immune evasion by SARS-CoV-2. It is noteworthy that many negative regulators of type-I IFN signaling were shown to be involved in cancer [80] and that immune-evasion also constitutes a hallmark of cancer [13].

Moreover, the most up-regulated lncRNA genes found in the infected samples—MYRF-AS1, ATP2A1-AS1, CTC-338M12.4, MESTIT1, PRANCR, and SLC25A5-AS1—are associated with several types of cancers in a conflicting manner. For instance, MYRF-AS1 was associated with genomic instability and is considered a risk factor in Non-small cell lung cancer [81], while PRANCR is up-regulated in primary ovarian cancer [82] and SCL25A5-AS1 was associated with the bladder cancer growth [83]. Conversely, ATP2A1-AS1 is considered a protective factor in cervical cancer [84], and CTC-338M12.4 is down-regulated in bladder cancer [85]. In turn, MESTIT1 is likely a protective factor in breast cancer [86] and a risk factor in prostate cancer [87].

Little is known about the long-term consequences of COVID-19 and its relationship with cancer development, although still largely elusive, cannot be excluded [88].

### 4.3. Coronavirus Disease

Another significantly enriched KEGG pathway was coronavirus disease (hsa05171). This pathway included up-regulated genes involved mainly in immune response and translation, with the co-occurrence of ANGII-ATR1R-Nox, RIG-I-IRF7/3, MDA5-IRF7/3, type-I IFN, TLR2/4-MAPK, TLR2/4-NF-κB, TNF-NF-κB, ribosome, and translation initiation signaling. Among the genes over-expressed in this pathway is the ACE gene. This gene encodes the angiotensin-converting enzyme (ACE), which converts angiotensin I (ANG I) to angiotensin II (ANG II) [89]. SARS-CoV-2 infects human cells mainly by the high-affinity binding of the S protein RBD to the ANG II-converting enzyme (ACE II), leading to its down-regulation. This process elicits the accumulation of ANG II and the overactivation of the ACE/ANG II/ANGII type 1 receptor (AT1R) axis [90]. This pathway is strongly pro-inflammatory and pro-oxidant, triggering the activation of NADPH oxidase, NF-κB, MAPK, and STAT1 signaling, which contributes to the expression of pro-inflammatory cytokines—like IL-1β, IL-6, IL-10, and TNF-α—and to cytokine storm [91]. Moreover, the ACE/ANG II/AT1R signaling induces the phosphorylation and enhancement of the ADAM-17 protease, which cleaves the ectodomain of several transmembrane proteins—including TNF-α and membrane-bound IL-6 receptor—and leads to their activation and release in a soluble form [92].

Several genes encoding ribosomal proteins, from both the 40S and 60S subunits, were significantly up-regulated in the coronavirus disease KEGG pathway, as well as in the WikiPathways cytoplasmic ribosomal proteins (WP477) and KEGG pathway ribosome (hsa03010), also enriched in our study (Figure 3B). Such proteins are involved in translation initiation in ribosomes, wherein a host cells’ process is shown to be hijacked by SARS-CoV-2. The NSP1 of SARS-CoV-2 prompts a translational shutdown of the host’s mRNA through the binding of its C-terminal domain to the mRNA entry channel at the ribosome 40S subunit, while enabling viral mRNA translation by interacting with a conserved region in the full-length SARS-CoV-2 5′UTR [93,94]. This process inhibits cellular antiviral defense mechanisms that rely on the expression of host factors, like the IFN response and RIG-I receptor signaling, and may facilitate efficient viral replication. Thus, the NS1-ribosome interaction is considered a candidate for structure-based drug design [94].

Immune-related genes, such as IRF3, ISG15, IκBKE, OAS1, MX1, and IL-6, were also significantly over-expressed in the hsa05171 pathway. Proteins encoded by these genes are involved in the RIG-I/MDA5-IRF7/3 (IRF3, ISG15, and IKBKE) and TLR2/4 (FOS, JUN, and NFKBIA) signaling pathways or expressed downstream of IFN-α/β (OAS1 and MX1), TLR2/4-NF-κB (IL-6), and ANGII-AT1R-NOX (IL-6) signaling. RIG-I- and MDA5-IRF7/3 signaling consist of the sensing of viral RNA by the cytoplasmic viral RNA sensors RIG-I and MDA5, which drives a cascade with the downstream phosphorylation and nuclear translocation of IRF3, which triggers type-I IFN production [95]. IFN-I, in turn, signals the activation of Janus Kinase 1 (JAK1) and Tyrosine Kinase 2 (TYK2), leading to the phosphorylation of STAT1 and STAT2, which form a heterodimer that, in association with IRF9, triggers the transcriptional activation of interferon-stimulated genes (ISGs) [96]. OAS1 and MX1 are ISGs with antiviral functions [97] and their transcription was up-regulated in our infected samples. Several SARS-CoV-2 proteins have been shown to antagonize IFN-I response at both RIG-I-/MDA5-IRF7/3 and IFN-α/β signaling levels [79]. For instance, NSP6 [96], NSP13 [96], and NSP9a [98] inhibit several steps of the phosphorylation cascade triggered by RIG-I and MDA5 signaling and, hence, hinder IFR3 phosphorylation. Likewise, ORF6 [96], NSP12 [99], and NSPs 13–15 [96] inhibit the nuclear translocation of IRF3; while NSPs 1 and 6 inhibit the phosphorylation of STAT1/STAT2 and suppress the type-I IFN-mediated ISGs expression [96]. Furthermore, Thoms [94] demonstrated the translational inhibition of the RIG-I and ISG15 expression mediated by the SARS-CoV-2 NSP1 in ribosomes. Our results showed a transcriptional up-regulation of genes belonging to pathways related to innate immune response that are antagonized by SARS-CoV-2 proteins [79]. However, the up-regulation of some ISGs—Mx1, OAS1, and ISG15—indicates that these pathways are not abrogated, despite the antagonism of the viral proteins. Nonetheless, these genes, even over-expressed at a transcriptional level, are perhaps inhibited by NSP1 at a translational level [94].

### 4.4. Other Immune-Related Pathways

Consistently, the RIG-I-like receptor signaling KEGG pathway (hsa04622) and the WikiPathways novel intracellular components of RIG-I-like receptor (RLR) pathway (WP3865) were also significantly enriched in infected vs. uninfected Calu-3 cells. Besides the aforementioned genes, these pathways also contain significantly up-regulated genes with regulatory functions, like NLRX1, TKFC, and PIN1. NLRX1—also belonging to the enriched WikiPathways SARS-CoV-2 mitochondrial interactions (WP5038)—encodes a protein located in the mitochondrial outer membrane that interacts with the mitochondrial antiviral signaling protein (MAVS) and disrupts the RIG-I-MAVS signaling, attenuating the downstream activation of IRF3. Hence, the NLRX1-MAVS interaction weakens the cytokine response to viral infections and prevents an overzealous immune response [100]. The TKFC gene encodes adihydroxyacetone kinase, which interacts with MDA5 and suppresses its antiviral signaling [101], while the PIN1 gene encodes a protein that associates with IFR3 and facilitates its ubiquitin-mediated proteasomal degradation [102].

Another significantly enriched immune-related KEGG pathway was the C-Type lectin receptor signaling (hsa04625). C-type lectins are pattern recognition receptors that recognize a range of carbohydrate structures—like mannose, fucose, sialic acid, and β-glucan—and are mainly expressed in myeloid cells, especially in macrophages and dendritic cells [103]. Among their numerous functions are pathogen sensing, the initiation of the immune response, and T helper (Th) cell differentiation [103,104]. In our work, the up-regulated genes belonging to the C-type lectin receptor pathways are mainly related to the Dectin-1 (PYCARD, NFKBIA, EGR2, and NFKB2) [105] and DC-SIGN (PKL3, MRAS, BCL3, and IKBEKE) [106,107] signaling. The former, upon stimulation by β-glycans, leads to the release of IL-1β, IL-6, IL-12, and IL-23, which forms a cytokine environment that skews the Th cell response towards a Th17 profile. The latter, upon stimulation by mannose, also produces a Th17-skewed cellular response, while, when stimulated by fucose, promotes Th2 and T follicular helper (Tfh) differentiation [103]. The Th17 cellular response is important in the defense response to fungus, promotes neutrophil migration to the sites of infection, and is associated with hyperinflammatory disorders [108]. In turn, the simultaneous activation of Th2 and Tfh responses by fucose leads to long-term humoral responses [109].

Among the genes up-regulated in the hsa04625 KEGG pathway are PYCARD, IL6, IKBKE, and BCL-3. PYCARD encodes an inflammasome-adaptor protein containing pyrin (PYD) and caspase recruitment (CARD) domains. It is involved in the assembly of the NLRP3 inflammasome and caspase-1 activation. These processes trigger the maturation of IL-1β and IL-18 [110] and promote pyroptosis by cleaving GSDMD [105]. PYCARD is also involved in non-canonical IL-1β maturation [111] and apoptosis [112] through caspase-8 activation. IL-1β is a strongly pro-inflammatory cytokine that exerts a range of inflammatory and antimicrobial activities, including the induction of fever and Th 17 differentiation [113]. The IL6 gene encodes IL-6, which prompts the differentiation of Th17 cells while suppressing the activation of Treg cells [114]. A skewing of T cell activation towards a Th17 phenotype was described in COVID-19 patients [115]. In turn, IKBKE encodes a kinase of the NF-κB inhibitor and is involved in the DC-sigh-mediated Th 2 differentiation following fucose biding. IKBKE suppresses the CYCD deubiquitinase activity that prevents the nuclear translocation of BCL-3, allowing for the BCL-3-mediated down-regulation of pro-inflammatory cytokines and the up-regulation of the IL-10 expression [107]. De Biasi et al. [115] observed a contradictory immune response in COVID-19 patients characterized by a marked plasma increase in IL-10 and other Th2 cytokines along with inflammatory and Th17-related cytokines.

The mucosal epithelium is immunologically active and functions as a regulator of innate and adaptive immune responses [116,117]. Indeed, human bronchial epithelial cells were shown to recognize house dust mites (HDMs) through β-glycan receptors, probably Dectin-1, and, as a response, secrete CCL20, a chemokine that attracts immature dendritic cells [118]. Murine bronchial epithelial cells, under stimulation by HDM and HDM + LPS, were also found to promote Th2 and Th17 differentiation of naïve T CD4+ cells [119]. Furthermore, DC-sigh is expressed by MUC1-producing type-II alveolar cells and binds to SARS-CoV-2 RBD [120]. Pathogens sensing by C-type lectin-receptors and the triggered downstream signaling pathways are glycan-type-dependent [103]. The SARS-CoV-2 S protein is a heavily glycosylated trimer with 22 canonical N-linked glycosylation sites per protomer and several o-glycosylation sites [121], whose glycan profiles are heterogeneous and host cell-dependent, varying from oligomannose to fucosylated glycans [121,122]. Hence, our results may indicate that infected Calu-3 cells were able to identify glycan moieties present in the SARS-CoV-2 glycoproteins through C-type lectin receptors and activate downstream signaling pathways that lead to the expression of cytokines and chemokine that promote the Th17 and Th2 differentiation of CD4+ lymphocytes.

Th17 cells produce IL-17, an inflammatory cytokine that mediates the recruitment of neutrophils and macrophages to infected tissues. Upon engagement with their receptors, IL-17 triggers signaling pathways that lead to the release of other pro-inflammatory cytokines and chemokines by several alveolar cell types, including macrophages, epithelial, and endothelial cells, which may contribute to cytokine storm and SARS [123]. In our work, the KEGG IL-17 signaling pathway (hsa04657) was also significantly enriched in infected vs. uninfected Calu-3 cells, and its up-regulated genes are mainly involved in MAPK [124] and NF-κB [125] signaling pathways and include both positive (NF-κB, JUN, FOS, FOSB, and MAPK15) [126] and negative (TRAF4, TNFAIP3, and NFKBI) [127,128] regulators, in addition to a product (IL6) of the transcriptional activation mediated by these pathways [123]. IL-17 is typically secreted by activated T CD4+ cells with a Th17 profile and is not over-expressed in our samples. However, a study showed the molecular mimicry of IL-17 by the SARS-CoV-2 ORF8 protein, which binds to several IL-17 receptors and activates downstream signaling pathways leading to the production of pro-inflammatory cytokines and chemokines [129]. Thus, our results suggest the activation of the IL-17 signaling pathway by the ORF8 protein. The enrichment of the KEGG IL-17 signaling pathway in SARS-CoV-2-infected Calu-3 was also described in a transriptomic study by Sun et al. [130].

In addition, the TNF signaling KEGG pathway (hsa04668) was also significantly enriched in our infected samples, which is in line with previous studies [130]. This pathway included mainly up-regulated genes involved in TNF-p38 (CEBPB, TRAF2, RPSGKA4, and CREB5), TNF-JNK (FOS, JUN, and TRAF2), TNF-NF-κB (TRAF2, and NFKBIA), and TNF-IRF1 signaling pathways, which lead to the release of pro-inflammatory cytokines and apoptosis [131,132]. Among the up-regulated genes expressed downstream of these pathways are IL6 and some negative regulators of TNF signaling, like SOCS3, BCL3, and TNFAIP3 [133].

Although the IL6 gene is not among the top 20 DEGs, it is noteworthy that it is over-expressed downstream to several signaling pathways, like TNF, IL17, C-type lectin receptors, and COVID-19 disease KEGG pathways. IL-6 levels are increased in COVID-19 patients as part of the cytokine storm [134] and have been considered as a biomarker for disease severity [135,136]. A study conducted by Blanco-Melo et al. [14] showed a transcriptional feature with a high expression of chemokines and IL-6 juxtaposed to reduced innate immune response and low levels of type I and III interferons in tissues from COVID-19 patients and 0.2 MOI-infected cells (Calu-3 and A549).

### 4.5. Metabolic Transcriptional Model and Limitations of the Study

The transcriptional characterization of infected vs. uninfected Calu-3 cells and the functional enrichment of the DEGs reveal that SARS-CoV-2 infection promotes a set of cellular responses characterized by the transcriptional activation of genes involved in antiviral and innate immune responses, inflammatory processes, pyroptosis and autoimmunity, cell proliferation, apoptosis, and mRNA translation (Figure 4). Several genes, especially those involved in immune and antiviral responses and mRNA translation, encode proteins that are antagonized by SARS-CoV-2 in a sort of immune-evasion and translational reprogramming of cellular functions. Among the up-regulated genes involved in immune response and inflammatory signaling, there are both positive and negative regulators. Moreover, despite a cancer cell line, Calu-3 cells have yielded transcriptional signatures that are largely reproducible in other cellular contexts, like in airway samples from COVID-19 patients [13].

Our results are in line with other transcriptome studies conducted in SARS-CoV-2-infected Calu-3 cells, which showed responses associated with IFN I, II, or III signaling [13,14,15,16,17]; innate immunity, inflammation, and defense against virus [13,14]; TNF and IL-17 signaling [130], and signaling mediated by RIG-I/MDA5 [13,16]. In contrast, chemokine genes are generally up-regulated in infected Calu-3 cells [13,14,15,17,130], which were not found in our study (Supplementary File S1). This transcriptional picture with the predominance of INF and chemokine genes and the enrichment of pathways associated with interferon response and innate immunity was also described for Calu-3 cells infected with other respiratory viruses, such as Rhinovirus (RV), Influenza A (IAV), and Influenza B (IBV) [137]. Moreover, a study conducted by Sun et al. [17] described a similar response for Calu-3 cells infected with either SARS-CoV-2 or SARS-CoV, which was not observed for the MERS-CoV-infected ones. Therefore, as reported by previous studies [13,14,15,16,17,137], our results describe cellular responses typical not only to SARS-CoV-2 but also to other viral infections. However, we found, as a novelty, a higher degree of activation for signaling pathways related to pyroptosis and autoimmunity, like NETs formation and STING pathway in Kawasaki-like disease and COVID-19, with the involvement of the GSDMD gene.

Nonetheless, our results describe transcriptional changes that took place 24 h post-infection. These changes are transient and may not reflect those that would occur at other time points [65]. In addition, infection with SARS-CoV-2 was performed at 0.5 MOI, which virtually means 0.5 functional virions delivered per cell, or 1 virion for every two cells [14]. Blanco-Melo et al. [14] described distinct transcriptional changes in SARS-CoV-2-infected cells and tissues according to the applied MOI, in which infection at 2 MOI induced stronger IFN-I and -III responses than 0.2 MOI-infection. Such differences in transcriptional responses according to infection methodology can make it difficult to directly compare different results. However, it is likely that culture infections performed at low MOI (<1) more closely mimic natural infection [138]. Another limitation of our study is the use of an in vitro approach with a lack of interplay with immune cells and other organ tissues, though it can de-convolute cell-specific responses [13].

## 5. Conclusions

RNA-seq was performed in Calu-3 cells to identify DEGs in SARS-CoV-2-infected vs. uninfected cells. The functional analysis of the DEGs observed 24 h postinfection showed the predominance of genes involved in innate immune response, antiviral signaling, inflammation, cell proliferation, apoptosis, and mRNA translation. These results reinforce some previous findings and point to the RNA-seq of SARS-CoV-2-infected Calu-3 cells as a reproducible and valuable method. In addition, responses related to pyroptosis and autoimmunity were also identified. These infection-induced transcriptional changes, although transient, may help to characterize the early stages of the cellular response to SARS-CoV-2 infection and elucidate COVID-19 pathogenesis at airway epithelium level, in addition to revealing potential biomarkers and possible drug targets. Early and late time periods should be considered in future studies to better characterize the cellular dynamics prompted by SARS-CoV-2 infection, which would be valuable to the understanding of COVID-19 pathogenesis. Our data must be validated by independent experiments at both mRNA and protein levels. Further comparisons with transcriptional data from in vivo experiments and COVID-19 patients, are needed for a deeper understanding of the results described here.

## Figures and Tables

**Table 1:** Top 20 differentially expressed genes in Calu-3 cells 24 h after SARS-CoV-2 infection (0.5 MOI).

Gene Symbol	ENSEMBL	Gene Name	Log2fc	Regulation
GCSAM	ENSG00000174500	Germinal center associated signaling and motility	−3.567	Down
CDRT1	ENSG00000241322	F-box and WD repeat domain containing 10B	−2.814	Down
EPHA4	ENSG00000116106	EPH receptor A4	2.631	Up
SUGCT	ENSG00000175600	Succinyl-CoA:glutarate-CoA transferase	−2.436	Down
ETV2	ENSG00000105672	ETS variant transcription factor 2	2.364	Up
H3C13	ENSG00000183598	H3 clustered histone 13	2.252	Up
HSPA6	ENSG00000173110	Heat shock protein family A (Hsp70) member 6	2.131	Up
SYCP3	ENSG00000139351	Synaptonemal complex protein 3	−2.058	Down
SNPH	ENSG00000101298	Syntaphilin	2.054	Up
SECTM1	ENSG00000141574	Secreted and transmembrane 1	1.942	Up
TAS1R3	ENSG00000169962	Taste 1 receptor member 3	1.886	Up
FOS	ENSG00000170345	Fos proto-oncogene, AP-1 transcription factor subunit	1.872	Up
GATD3	ENSG00000160221	Glutamine amidotransferase class 1 domain containing 3	1.855	Up
ANKRD24	ENSG00000089847	Ankyrin repeat domain 24	1.814	Up
EGR2	ENSG00000122877	Early growth response 2	1.793	Up
TEAD2	ENSG00000074219	TEA domain transcription factor 2	1.786	Up
GP6	ENSG00000088053	Glycoprotein VI platelet	−1.780	Down
MMP17	ENSG00000198598	Matrix metallopeptidase 17	1.780	Up
H2AC13	ENSG00000196747	H2A clustered histone 13	1.771	Up
SLC34A1	ENSG00000131183	Solute carrier family 34 member 1	1.756	Up

## Data Availability

The raw RNA-seq data that supports the results obtained in this study is openly available at BioProject: PRJNA993611: Transcriptome of SARS-CoV-2 Calu-3.

## Supplementary Materials

The following supporting information can be downloaded at: https://www.mdpi.com/article/10.3390/pathogens12111373/s1, Supplementary File S1: Transcripts identified in Calu-3 cells. Supplementary File S2: Principal Component Analysis.
