# Supplementary material for: Transcriptional Profiling of SARS-CoV-2-Infected Calu-3 Cells Reveals Immune-Related Signaling Pathways
Source: Pathogens. 2023 Nov 20;12(11):1373. doi: 10.3390/pathogens12111373 (PMC10674242; doi:10.3390/pathogens12111373)
Supplement: Supplementary file 1 [file pathogens-12-01373-s001.zip › Supplementary file S2.pdf]

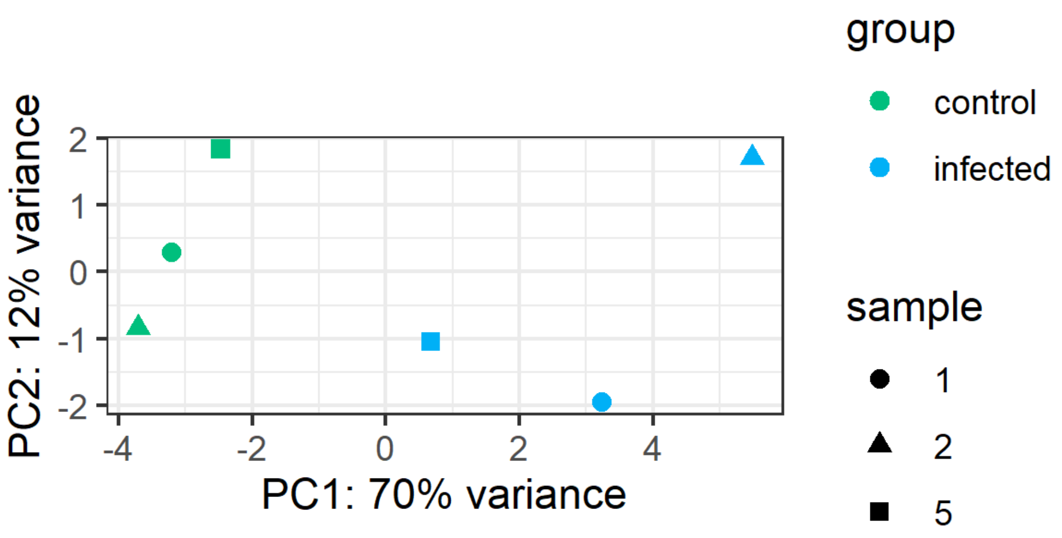

Supplementary file S2 - Principal Component Analysis (PCA). PC1: Principal Component; PC2: Principal Component 2. Green: control samples (C1, C2, and C5). Blue: infected samples (I1, I2, and I5).
